# Analysis of *NPM1* splice variants reveals differential expression patterns of prognostic value in acute myeloid leukemia

**DOI:** 10.18632/oncotarget.19871

**Published:** 2017-08-03

**Authors:** Malgorzata Zajac, Anna Dolnik, Grazyna Stasiak, Joanna Zaleska, Michal Kielbus, Jakub Czapinski, Matthias Schunn, Stephany C. Correa, Eliza Glodkowska-Mrowka, Reddy Chakkarappan Sundaram, Olga Jankowska-Lecka, Richard F. Schlenk, Hartmut Döhner, Konstanze Döhner, Andrzej Stepulak, Lars Bullinger, Krzysztof Giannopoulos

**Affiliations:** ^1^ Department of Experimental Hematooncology, Medical University of Lublin, Lublin, Poland; ^2^ Department of Internal Medicine III, University of Ulm, Ulm, Germany; ^3^ Department of Biochemistry and Molecular Biology, Medical University of Lublin, Lublin, Poland; ^4^ Postgraduate School of Molecular Medicine, Medical University of Warsaw, Warsaw, Poland; ^5^ Stem-Cell Laboratory, Bone Marrow Transplantation Unit, National Cancer Institute (INCA), Rio de Janeiro, Brazil; ^6^ Department of Immunology, Medical University of Warsaw, Warsaw, Poland; ^7^ Department of Laboratory Diagnostics and Clinical Immunology of Developmental Age, Medical University of Warsaw, Warsaw, Poland; ^8^ Department of Hematooncology and Bone Marrow Transplantation Unit, Medical University of Lublin, Lublin, Poland; ^9^ Department of Hematology, St. John's Cancer Center, Lublin, Poland

**Keywords:** AML, splicing variants, NPM1

## Abstract

Mutations of the nucleophosmin-1 (*NPM1*) gene in cytogenetically normal (CN) acute myeloid leukemia (AML) identify a group of patients with more favorable prognosis. *NPM1* encodes three main alternatively spliced isoforms R1(B23.1), R2(B23.2), and R3(B23.3). The expression of splice variants R1, R2 and R3 were higher in AML patients compared to normal cells of healthy volunteers (HVs), although RNA-seq analysis revealed enhanced R2 expression also in less differentiated cells of HVs as well as in AML cells. The variant R2, which lacks exons 11 and 12 coding for the nucleolar localization domain, might behave similar to the mutant form of *NPM1* (*NPM1*mut). In accordance, in CN-AML high R2 expression was associated with favorable impact on outcome. Moreover, functional studies showed nucleolar localization of the eGFP-NPM1 wildtype and cytoplasmic localization of the eGFP-NPM1 mut protein. While the eGFP-NPM1 R2 splice variant localized predominantly in the nucleoplasm, we also could detect cytoplasmic expression for the R2 variant. These results support a unique biological consequence of R2 overexpression and in part explain our clinical observation, where that high R2 variant expression was associated with a better prognosis in CN-AML patients.

## INTRODUCTION

Recently, next generation sequencing technology has identified many new gene mutations in acute myeloid leukemia (AML) that provide novel insights into the mechanisms of leukemogenesis and that further unravel the molecular heterogeneity, in particular within the group of cytogenetically normal (CN) AML [1, 2]. In addition to genomic abnormalities, aberrant expression levels of several genes have been identified as prognostic markers [3, 4], so as deregulated gene expression also involved in CN-AML pathogenesis. However, underlying mechanisms are still poorly understood and require more detailed characterization in order to define novel markers of improved leukemia risk stratification.

In addition, mRNA splicing has been reported to be involved in human disease development, and many cancer-related genes have been shown to be regulated by alternative splicing [5]. In accordance, in myeloid disease several reports have revealed mutations in genes encoding splicing factors, such as *SF3B1* [6, 7], and recently a heterogeneous genomic category of AML with mutations in genes encoding chromatin and RNA-splicing regulators, accounting for 18% of patients, could be identified [8]. Moreover, first analyses of alternative splicing in bone marrow of AML samples revealed a number of significantly spliced genes, which encode e.g. proteins such as NOTCH2, CD13 or FLT3 [9].

Recently, *NPM1* mutations have been defined from a provisional to an established entity within the WHO classification of AML [10, 11]. This new entity bears distinct genetic, pathological and clinical features [12, 13]. Of particular importance is the fact that mutations in *NPM1* without concomitant FMS-like tyrosine kinase 3-internal tandem duplication (*FLT3*-ITD) mutations identify a group of CN-AML patients with favorable prognosis [12, 14, 15]. NPM1 (also known as B23) was first identified as a multifunctional phosphoprotein, which shuttles between nucleus and cytoplasm. It participates in ribosome biogenesis, supporting cell growth and proliferation [16–18]. NPM1 physically interacts with many cellular proteins, including the tumor suppressors p53 and ARF. For example, NPM1 stabilizes the oncosuppressor ARF and determines its subcellular localization, thus contributing to modulating growth-suppressive pathways [19–21].

*NPM1* mutations occur specifically in about 30% of adult *de novo* AML patients. These mutations cause delocalization and destabilization of ARF. Absence of NPM1 in the nucleus in consequence may suppress the antioncogenic effect of the ARF-MDM2-p53 signaling pathway. *NPM1* mutant AML presents with a specific gene expression profile and a specific microRNA (miRNA) signature [22, 23]. In accordance, the relocation of NPM1 into the cytoplasm was shown to cause overexpression of a number of *HOX* genes potentially involved in hematopoietic stem cell differentiation. Overexpression of *HOXA4*, *HOXA6*, *HOXA7*, *HOXA9*, *HOXB9* and *MEIS1* were observed in *NPM1* mutated cells, as well as AML with *MLL* abnormalities. However, it was also found that some of *HOX* genes like *HOXB2*, *HOXB3*, *HOXB5*, *HOXB6* and *HOXD4* are upregulated only in cells with *NPM1* mutation. However, the mechanism by which *NPM1* affects *HOX* gene expression is still unclear [24].

*NPM1* mutations are usually associated with CN karyotype (85%), while the remaining 15% of patients mostly carry minor chromosomal abnormalities that are thought to be a secondary events [25]. It was found that *NPM1* mutations correlate with some driver molecular events like *FLT3*-ITD, *DNMT3A,* *IDH1*, *IDH2* and *TET2* mutations [1, 8, 26], while *NPM1* mutations are mutually exclusive or rarely co-occur in *MLL*-PTD, *RUNX1*, *CEBPA* and *TP53* mutant cases [2].

The *NPM1* gene contains 12 exons and in humans maps to chromosome 5q35. It encodes at least three main alternatively spliced isoforms: R1 (B23.1), R2 (B23.2), and R3 (B23.3). The prevalent isoform R1 is translated from exon 1 to 9 and 11 to 12, the isoform R2 contains exons 1 to 10, and little information is available on the R3 isoform lacking exons 8 and 10 [27, 28]. Of special interest is isoform R2, which lacks the exons coding the domain responsible for the nucleolar localization. Due to the lack of exons 11 and 12, this isoform preferentially localizes in the nucleoplasm [29] that might affect signal pathways influencing thereby patients outcome or modulating treatment response.

NPM1 is also frequently overexpressed in solid tumors of different histological origin (such as gastric, colon, ovarian, and prostate carcinomas) [30–33], however to date *NPM1* mutations seem to be exclusively found in AML patients, although the relevance of splice variant expression remains undetermined. Since splice variants play an important role in cellular functioning and splicing factor mutations have been reported in myeloid tumors including AML [8, 34], the current study focuses on the characterization of *NPM1* splice variants expression as well as its impact in AML patients.

## RESULTS

### Expression levels of *NPM1* splice variants in AML patients

In the first cohort of 104 patient samples qRT-PCR was performed for three splice variants of *NPM1* gene. The following *NPM1* splice variants were evaluated: R1 translated from exon 1 to 9 and 11 to 12, R2 containing exons 1 to 10, and R3 lacking exons 8 and 10. *NPM1* splice variant and primer locations are presented in Supplementary Figure 1A. As a control we isolated RNA from peripheral blood mononuclear cells (PBMC) of six healthy volunteers (HVs) and measured expression of *NPM1* splice variants in this group. The clinical, cytogenetic and molecular genetic characteristics of the patients in this study are summarized in Table 1.

**Table 1:** Baseline characteristics of 201^*^ AML patients

Characteristics	All patients (n=201)	CN-AML patients (n=105)	AML patients with cytogenetic aberrations (n=92)
**Sex - no. (%)**			
Male	110 (55)	59 (56)	48 (52)
Female	91 (45)	46 (44)	44 (48)
**Age - yr**			
Median	49	49	48
Range	18-74	24-74	18-68
**Bone marrow blasts**			
Median - %	80	80	80
Range - %	0-100	6-100	0-100
Data not available – no. (%)	6 (3)	1 (0.5)	5 (5)
**Peripheral blood blasts**			
Median - %	57	48	65
Range - %	0-100	0-100	0-100
Data not available – no. (%)	6 (3)	1 (0.5)	5 (5)
**Type of AML - no. (%)**			
Primary AML	179 (89)	95 (90)	80 (87)
s-AML	10 (5)	6 (6)	4 (4)
t-AML	7 (4)	1 (1)	6 (7)
o-AML	5 (2)	3 (3)	2 (2)
***NPM1*** **mutations - no. (%)**	60 (30)	55 (52)	4 (4)
Data not available	6 (3)	0 (0)	6 (7)
***FLT3*-IDT mutations - no. (%)**	50 (25)	36 (34)	14 (15)
**Cytogenetic group - no. (%)**			
normal karyotype	105 (52)	105 (100)	16 (17)
complex karyotype	16 (8)		13 (14)
inv(16)	13 (6)		4 (4)
t(9;11) other	4 (2)23 (11)		23 (25)10 (11)
t(15;17)	10 (5)		14 (15)
t(8;21)	14 (7)		

^*^ For 4 patients data were not available. s-AML - secondary AML, t-AML - therapy related AML, o-AML - patients with a clinical history an oncological disease, which was not treated with chemotherapy or radiation therapy and therefore cannot be classified as t-AML.

The expression of splice variants R1, R2 and R3 were higher in all AML patients compared to HVs with a median 1.73 vs 0.55 (p=0.014), 0.98 vs 0.33 (p=0.093), and 2.54 vs 0.11 (p<0.001), respectively (Supplementary Figure 1B-1D).

### RNA-seq of AML and subpopulations of HVs cells

**Figure 1:**
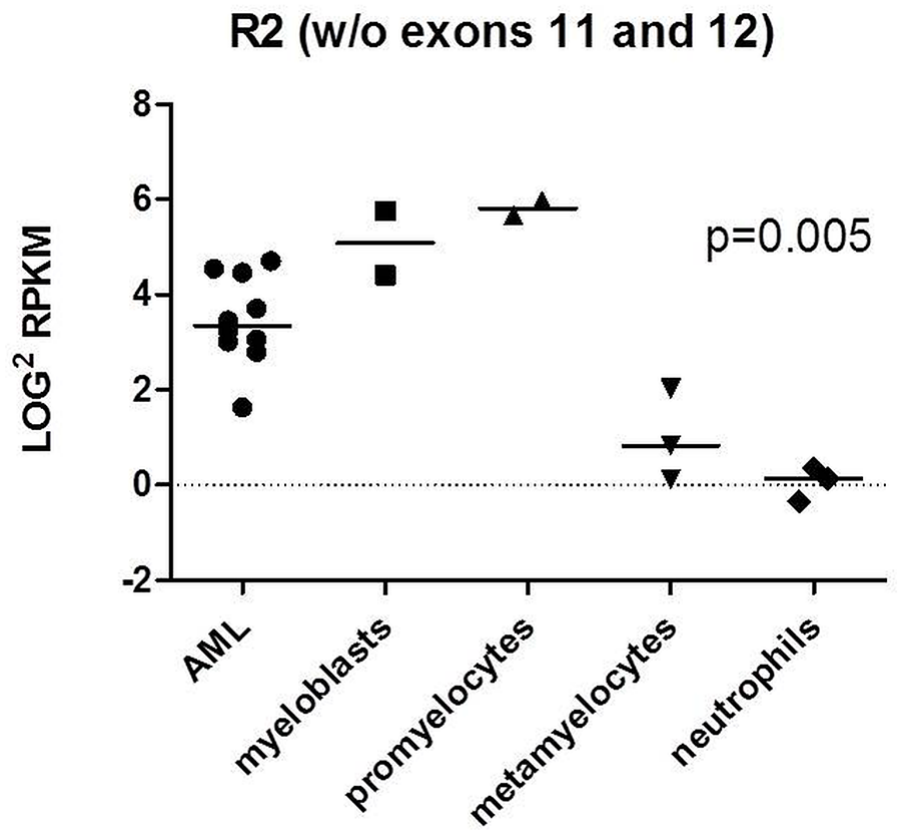
RNAseq analysis of R2 expression in 10 AML transcriptomes and 3 HVs bone marrow samples Ten cell fractions of cells from normal bone marrow were obtained: myeloblasts (from n=2 healthy donors), promyelocytes (n=2), metamyelocytes (n=3) and neutrophils (n=3). P-value refers to all analyzed subfractions of cells. The analysis was performed with ANOVA test.

As the relative expression should be assessed using normal stem cells from HVs, we performed RNA-seq analyses of FACS-sorted bone marrow samples of three HVs and ten transcriptomes of CN-AML cases. In total ten cell fractions of cells from normal bone marrow were obtained: myeloblasts (from n=2 healthy donors), promyelocytes (n=2), metamyelocytes (n=3) and neutrophils (n=3) [35], next R2 expression was assessed. We observed that the expression of R2 was significantly higher in less differentiated hematopoietic cells (such as myeloblasts, promyelocytes) and in AML blasts compared to more differentiated cells (metamyelocytes and neutrophils (p=0.005)) what can suggest R2 accumulation in blast cells (Figure 1).

### *NPM1* splice variant expression in CN-AML patients

To investigate whether *NPM1* expression is influenced by its mutational status, we separated the 52 CN-AML patients (from the first cohort of 104 patients) based on underlying *NPM1*mut or *NPM1* wildtype status (*NPM1*wt). Expression of the R2 splice variant tended to be elevated in *NPM1*mut compared to *NPM1*wt with a median expression of 0.78 vs 0.40, p=0.052, but there were no significant differences in R1 and R3 mRNA levels between *NPM1*mut and *NPM1*wt (0.97 vs 0.80, p=0.20 and 1.51 vs 2.40, p=0.64, respectively) (Supplementary Figure 2A-2C).

### Expression of NPM1 proteins

**Figure 2:**
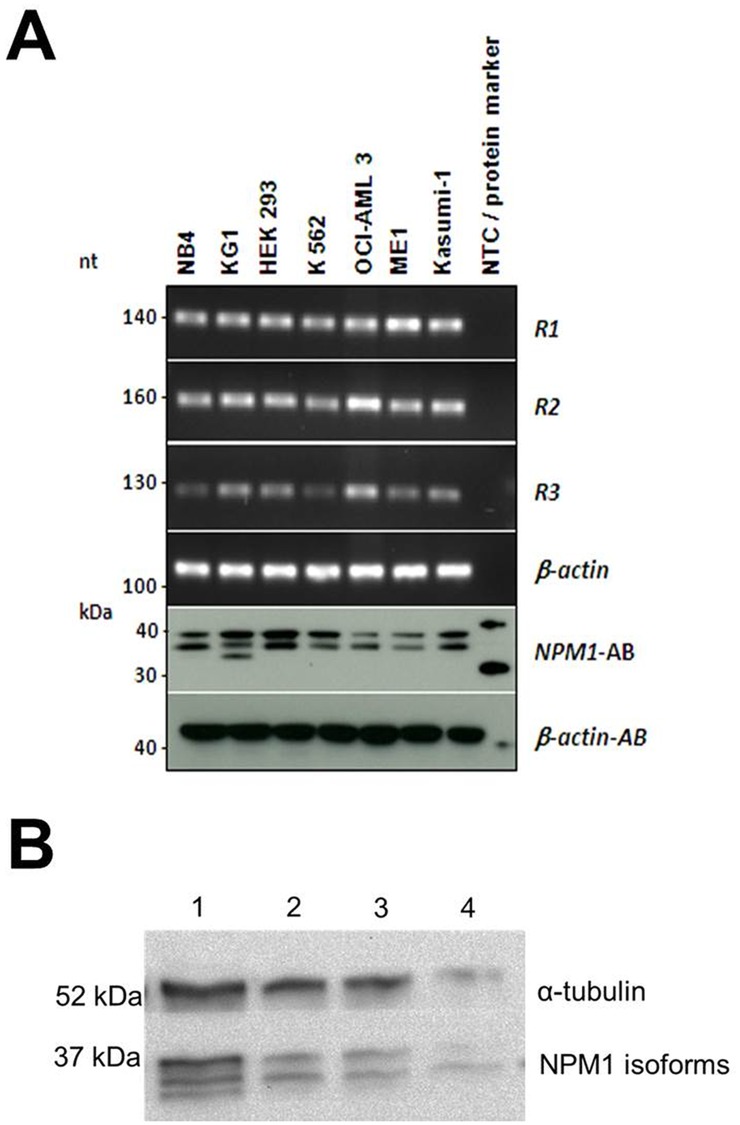
(A) Conventional PCR and Western blot analysis of NPM1 isoforms in cell lines cDNA from the cell lines NB4, KG1, HEK293, K562, OCI-AML3, ME-1 and Kasumi-1 was analyzed by conventional PCR and protein lysates of the corresponding cell lines were analyzed on Western blot. β-actin was used as a loading control. Conventional PCR shows the expression of R1, R2 and R3 *NPM1* isoforms in the cell lines, which could be confirmed by Western blot analysis. **(B)** Western blot analysis of NPM1 expression in 4 AML patients.

To evaluate the translation of the splice variants on the protein level, we performed Western blot analysis in selected cancer cell lines which revealed differential expression of NPM1 splice variants in leukemic myeloid cells (Figure 2A). While all cell lines seem to express the R2 variant, additional splice variant seems to exist as highlighted by the additional band in the KG1 cell line. In primary AML patient samples with available material for Western blot analysis, we also evaluated the expression of R2 as its expression might have prognostic impact. Western blot analysis of 3 AML patients demonstrates R1 and R2 expression at the protein level. In one patient we observed all 3 isoforms of NPM1 (Figure 2B).

### Impact of *NPM1* splice variant expression on outcome

Correlation of *NPM1* splice variant expression with overall survival (OS), event-free survival (EFS), and relapse-free survival (RFS) was evaluated by Kaplan-Meier analysis for groups with high and low expression of each splice variant (data were dichotomized at the median expression). High R2 splice variant expression was significantly associated with longer OS, EFS and RFS in the first data set of 104 AML patients (854 vs 403 days, p=0.019, 281 vs 182 days, p=0.034, and median not reached (n.r.) vs 323 days, p=0.014, respectively) (Supplementary Figure 3). We also found that high expression of R1 impacted OS, but not EFS and RFS (814 vs 402 days, p=0.029, 260 vs 205 days, p=0.29 and 1312 vs 345, p=0.23). We found no association with OS, EFS and RFS in case of high or low R3 expression (435 vs 669 days, p=0.83, 197 vs 288 days, p=0.26, and 471 vs 627 days, p=0.74) in 104 AML patients.

### Evaluation of R2 expression in a large AML cohort (n=201 cases)

**Figure 3:**
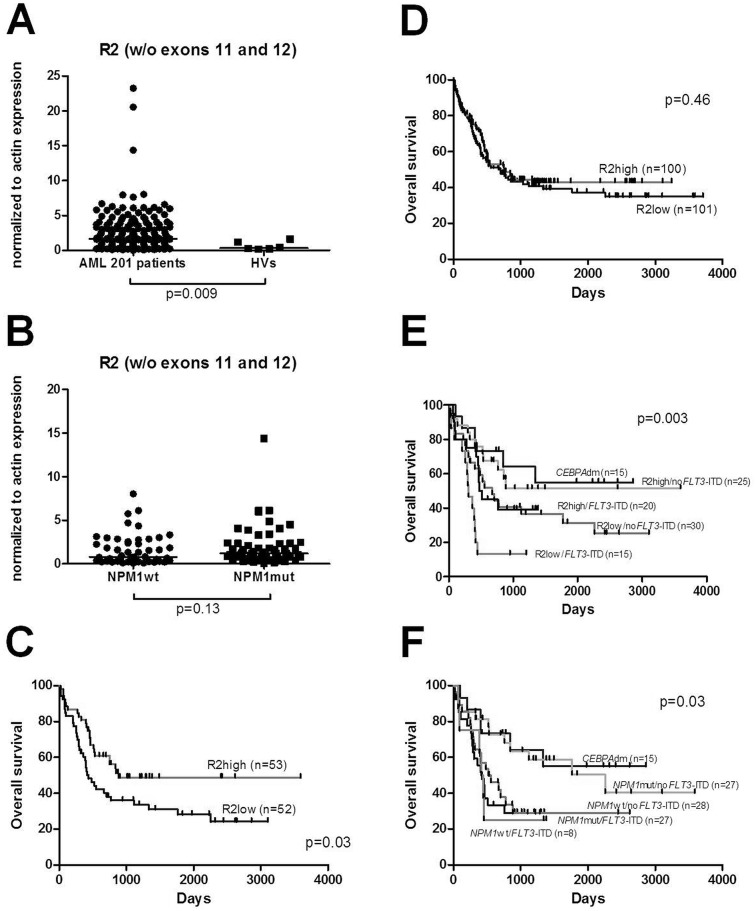
**(A)** Enhanced expression of *NPM1* splice variant R2 was observed in 201 AML patients compared to HVs. **(B)** Expression levels of *NPM1* splice variant R2 within CN-AML groups with *NPM1* mutations (*NPM1*mut) and without its mutations (*NPM1*wt). No difference was seen between those groups of patients. **(C)** OS in 105 CN-AML patients divided according to the expression levels of R2 splice variant. **(D)** OS of the total 201 AML patients divided according to the expression levels of R2. **(E)** OS in CN-AML patients divided into four groups according to the expression levels of R2 and *FLT3*-ITD mutational status: R2high/no*FLT3*-ITD R2low/no*FLT3*-ITD, R2low/*FLT3*-ITD, R2high/*FLT3*-ITD and *CEBPA*dm cases. **(F)** OS in CN-AML patients divided into four groups according to the *NPM1* and *FLT3*-ITD mutational status: *NPM1mut/noFLT3*-ITD, *NPM1wt/noFLT3*-ITD, *NPM1wt/FLT3*-ITD, *NPM1mut/FLT3*-ITD with additional group of *CEBPAdm*.

Based on these findings suggesting a biological and clinical role for the R2 splice variant, we decided to extend our cohort by an additional 97 AML patients in order to obtain a larger AML cohort (n=201) that will allow meaningful subgroup analyses. First, we confirmed that the expression of splice variant R2 was significantly higher in all AML patients (n=201) compared to HVs with a median expression of 1.64 vs 0.33 (p=0.009) (Figure 3A). While in the first cohort of CN-AML expression of R2 tended to be elevated in *NPM1*mut compared to *NPM1*wt, in the entire cohort of CN-AML (n=105) we found a similar trend between these groups (1.21 vs 0.82; p=0.13, respectively) (Figure 3B).

In the combined data set, high R2 splice variant expression was also associated with longer OS when CN-AML patients (n=105) were considered (880 vs 438 days, p=0.03) (Figure 3C), but there was no significant impact on OS in case of high or low R2 expression across all AML patients (n=201) (756 vs 669; p=0.46) (Figure 3D). We analyzed the relevance of R2 high expression and *FLT3*-ITD mutations in CN-AML by comparing R2high/no*FLT3*-ITD, R2low/no*FLT3*-ITD, R2low/*FLT3*-ITD, R2high/*FLT3*-ITD. From these four groups we were able to discriminate a group with the most favorable prognosis: R2high/no*FLT3*-ITD (median survival n.r.) and a group with the worst prognosis: R2low/*FLT3*-ITD (median survival 306 days).

Finally, we reflected our R2 results with regard to the molecularly defined CN-AML groups *NPM1*mut/no*FLT3*-ITD, *NPM1*wt/no*FLT3*-ITD, *NPM1*wt/*FLT3*-ITD and *NPM1*mut/*FLT3*-ITD. In our cohort, survival differences between these groups were smaller than between groups stratified according to R2 expression combined with *FLT3*-ITD mutational status (Figure 3E-3F). The most favorable group according *NPM1*/*FLT3*-ITD stratification was *NPM1*mut/no*FLT3*-ITD (median survival 2256 days) while *NPM1*mut/*FLT3*-ITD represented a group with the worst prognosis (median survival 423 days).

In accordance, in the CN-AML patient cohort univariate analysis revealed R2high/no*FLT3*-ITD genotype (HR, 0.507; 95%-CI, 0.275 to 0.936; p=0.03), *NPM1*mut/no*FLT3*-ITD genotype (HR, 0.515; 95%-CI, 0.273 to 0.97; p=0.004), *FLT3*-ITD alteration (HR, 1.987; 95%-CI, 1.187 to 3.325; p=0.009), high WBC (HR, 1.007; 95%-CI, 1.002 to 1.013; p=0.006), and age (HR, 1.054; 95%-CI, 1.028 to 1.08, p<0.001), but not gender, *CEBPA*, *NRAS*, *FLT3*-TKD or *NPM1* mutations, as significant variables for OS (Table 2). In multivariate analysis including known risk factors (age, WBC, *NPM1*mut/no*FLT3*-ITD genotype, *FLT3*-ITD mutations), in our cohort of CN-AML patients only *FLT3*-ITD mutations and age represented independent factors associated with shorter OS (HR, 1.954; 95%-CI, 1.167 to 3.271; p=0.011 and HR, 1.054; 95%-CI, 1.028 to 1.081; p<0.001, respectively) (Table 2).

**Table 2:** Univariate and multivariate analyses of overall survival (OS) in the entire cohort of 105 CN-AML patients

Variable	Univariate		Multivariate	
	HR (95% CI)	p	HR (95% CI)	p
R2high/noFLT3-ITD, vs other genotypes	0.51 (0.28-0.94)	0.03	0.69 (0.35-1.37)	0.29
Gender, male vs female	1.04 (0.63-1.71)	0.89		
Age	1.05 (1.03-1.08)	<0.001	1.05 (1.03-1.08)	<0.001
WBC	1.01 (1.00-1.01)	0.006	1.00 (0.99-1.00)	0.125
*NPM1* mutation, present vs absent	0.91 (0.56-1.50)	0.72		
*NPM1*mut/no*FLT3*-ITD, vs other genotypes	0.52 (0.27-0.97)	0.04	0.63 (0.31-1.27)	0.19
*CEBPA* mutation, present vs absent	0.48 (0.21-1.12)	0.089		
*FLT3-*ITD mutation, present vs absent	1.99 (1.19-3.33)	0.009	1.95 (1.17-3.27)	0.011
*FLT3-*TKD mutation, present vs absent	0.24 (0.03-1.76)	0.16		
*NRAS* mutation, present vs absent	1.86 (0.95-3.61)	0.067		

### Subcellular localization of NPM1 wildtype, R2 isoform and NPM1 mutant

**Figure 4:**
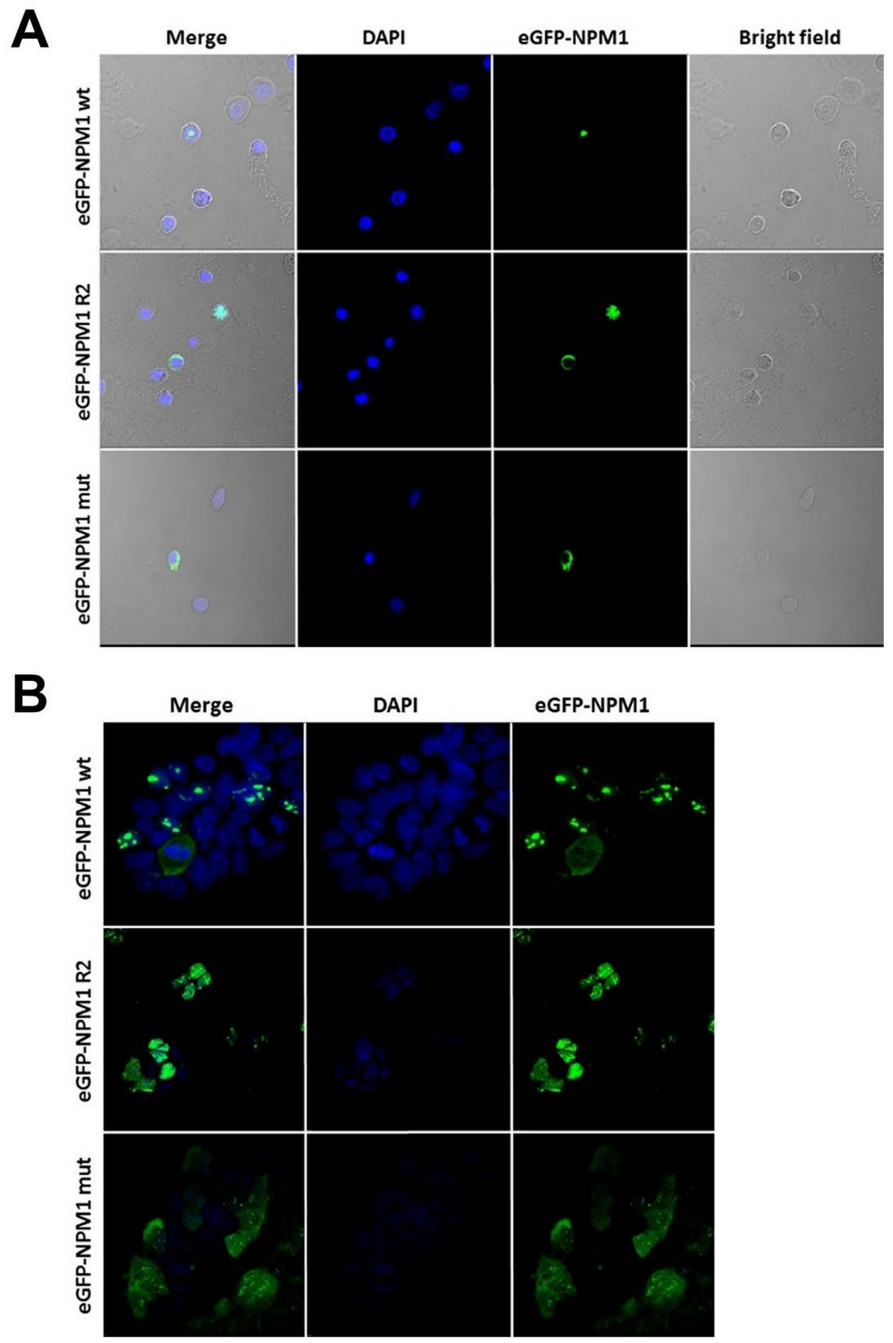
NPM1 wt, R2 and mut show different subcellular localization in AML patient and HEK293 cells **(A)** Confocal analysis of AML patient cells transiently transfected with expression vectors for eGFP-NPM1 wt, eGFP-NPM1 R2 or eGFP-NPM1 mut. Transfected cells expressed eGFP-NPM1 fusion proteins (green fluorescence). **(B)** Confocal analysis of the stable HEK293 cell lines expressing NPM1 variants (NPM1 wt, NPM1 R2 and NPM1 mut) fused with fluorescent GFP tag. Nuclei were stained with DAPI. Images were collected with Nicon Ti confocal microscope.

To test subcellular localization of the NPM1 wildtype, R2 isoform and NPM1 mutant, primary AML patient cells and HEK293 cells were transfected with expression vectors encoding the wildtype, R2 and mutated variant of NPM1 tagged with eGFP. Confocal microscopy showed nucleolar localization of the eGFP-NPM1wt protein and the eGFP-NPM1mut was localized in the cytoplasm, while the R2 splice variant exhibited variable subcellular localization. In AML patient cells eGFP-NPM1 R2 was detected in the nucleoplasm or cytoplasm, whereas in HEK293 cells the R2 variant showed only nucleoplasmic localization (Figure 4A-4B).

## DISCUSSION

The recent findings of frequent mutations affecting the splicing pathway in myelodysplastic syndrome (MDS) [6, 7] further highlight the importance of the mechanism of alternative splicing, which has been long associated with the development of cancer [5, 36]. Notably, whole exome sequencing of MDS samples identified recurrent mutations in multiple components of the RNA splicing machinery (such as *U2AF35, SF3B1, SRSF2* or *ZRSR2*) [6, 37]. The respective genes were found also mutated in ~26% of therapy-related AML or AML with MDS-related changes, and in ~7% of *de novo* AML cases [6]. Moreover, in a previous study we could show novel splicing-related mutations, which affected 10% of AML patients in a mutually exclusive manner, thereby pointing to an important role in the molecular pathogenesis of AML [34], which could recently be confirmed in a large AML targeted resequencing study [8]. In the current study, we now determined the expression pattern of *NPM1* splice variants in a well-defined cohort of 104 and 97 AML cases. Since *NPM1* splice variants have not been extensively studies so far, we focused our interest on the three main splice variants of *NPM1*. As *NPM1* encodes a protein important and frequently mutated in AML development, the expression of its modifications might also be involved in the pathogenesis of AML [38].

Recently, genome-wide microarray analysis using Exon arrays discovered genes significantly spliced in AML [5]. These studies identified novel splice variants specific for AML patients in comparison to normal cells such as *NOTCH2*, *CD13* and *FLT3* [9], but also splice variants discriminating leukemia subgroups such as *MAPK15* and *PLXNB1* [39]. Moreover, evidence was provided that the AML specific “splicing profile” was normalized in remission and recurred with patient relapse [5], thereby supporting a role of deregulated splice variants in the process of leukemogenesis, although many changes observed in this study might also reflect differences in the differentiation of cells.

Implicated in promoting cell growth NPM1 expression increases in response to mitogenic stimuli and above-normal amounts are detected in highly proliferating and malignant cells [40]. In accordance, NPM1 has been reported to be overexpressed at the protein level in various solid tumors [30–33] and in some cases it has been proposed as a cancer-specific marker. Tsui and colleagues [41] showed that the overexpression of *NPM1* at the mRNA level is independently associated with the recurrence of bladder carcinoma and progression to the more advanced stage. In our study we found that the expression levels of the splice variants R1, R2 and R3 of the *NPM1* gene were elevated compared to HVs suggesting that altered expression of *NPM1* splice variants might play a role in the process of tumorigenesis. Moreover, our RNA-seq analysis of certain subpopulation of HVs cells revealed R2 accumulation in less differentiated hematopoietic cells and in blast cells of AML.

With regard to CN-AML subgroups, our study demonstrated a favorable impact of high expression of the R2 splice variant on outcome. From the entire group of CN-AML patients, those with high R2 expression had a significantly longer OS compared to patients with low R2 expression levels. Similarly, our results suggest that the expression of R2 may allow the dissection of CN-AML patients into prognostically different subgroups. As the R2 splice variant represents a truncated form of the *NPM1* gene lacking exons 11 and 12 (coding for the domain responsible for nucleolar localization of the protein), this isoform is mostly localized in the nucleoplasm and/or cytoplasm [29] and thus might also have a biological impact in the malignant cells. Therefore, R2 might interact with nuclear proteins affecting signal pathways and thereby have an impact on the biology of the disease, which in turn is reflected in differences in treatment response and outcome. As the localization of the protein seems to be crucial for its functioning, we evaluated the impact of *NPM1*wt, R2 variant and *NPM1*mut expression in HEK293 and primary AML patient cells. Confocal microscopy showed nucleolar localization of the eGFP-NPM1wt protein, whereas the eGFP-NPM1mut was localized in the cytoplasm. The question is now, whether the subcellular localization of the R2 splice variant in the nucleoplasm and/or cytoplasm compared to the nucleolar localization of the wt variant does also contribute to altered gene expression.

While *NPM1* mutations have recently been shown to be also associated with differential expression of miRNAs [42, 43] aberrant expression of *NPM1* splice variants might in turn affect its translational regulation via miRNAs in AML.

In recent years several studies described a higher frequency of *FLT3* mutations in the group of *NPM1*mut, suggesting a possible pathogenic link between these mutations [12, 14, 38], and patients without concomitant *FLT3*-ITD were shown to have a better prognosis [12, 14, 15, 38]]. In line with the *NPM1* mutational status in our study survival curves demonstrated only a favorable impact of R2 expression in patients without *FLT3*-ITD. Notably, in our cohort survival differences seen between molecular mutation-defined groups according to a *NPM1*/*FLT3*-ITD stratification were less pronounced than between groups stratified according to R2 expression combined with *FLT3*-ITD mutational status. While we of course only studied a limited number of cases, based on our observations nevertheless additional studies are warranted to further evaluate the impact of R2 splice variant in AML. Our findings suggest that the analysis of splice variants might be added to the risk-classification of AML and could be also explored as novel biomarkers. With that regard, novel technological advances such as RNA-seq will provide valuable novel insights [44].

In summary, the expression of *NPM1* splice variants might be of biological importance in AML, especially in CN-AML patients, and future studies will have to further explore the prognostic value of the R2 splice variant expression in the light of the genomic AML landscape.

## MATERIALS AND METHODS

### Patient samples

Two independent cohorts of peripheral blood and/or bone marrow samples from adult Caucasian AML patients at diagnosis were provided by the German-Austrian AML Study Group (AMLSG) with patient informed consent and institutional review board approval from all participating centers. The first data set comprised 104 samples (including 52 CN-AML cases) from the AML HD98A (n=90, NCT00146120) and HD98B (n=14, NCT00151242) trials, and the second cohort contained 97 samples (including 53 CN-AML cases) from the AMLSG 07-04 study (NCT00151242). In the entire cohort of 201 patients samples 162 samples were obtained from bone marrow (bone marrow mononuclear cells, BMMC) and when bone marrow was not reached samples were taken from peripheral blood (peripheral blood mononuclear cells, PBMC) (the rest of 39 samples). As these two groups represent different compartments of cellular composition we performed R2 expression evaluation in both groups to see if there is significant difference between them. We have not observed any changes in R2 expression between BMMC and PBMC groups (median 1.78 vs 1.52, p=0.66) (Supplementary Figure 1E).

Peripheral blood samples were taken from six healthy volunteers (HVs) with informed consent with respect to the use of their samples for scientific purposes.

### Cell isolation

PBMCs or BMMCs were isolated by Ficoll (Biochrom AG, Berlin, Germany) density gradient centrifugation. The viability of MCs was always >80%, as determined by trypan blue staining. The viable cells were quantified in a Neubauer chamber (Zeiss, Oberkochen, Germany) and stored for RNA isolation in liquid nitrogen.

### mRNA preparation and reverse transcription

For the isolation of mRNA from PBMCs and BMMCs, the QIAamp RNA Blood Mini Kit (Qiagen, Venlo, Netherlands) was used according to the manufacturer's instructions and RNA quality were assessed by gel electrophoresis. Reverse transcription was done with SuperScript III First-Strand Synthesis System for RT-PCR (Invitrogen, Life Technologies Corporation, Carlsbad, CA, USA) using random hexamers and following the manufacturer's protocol. One μg of RNA was reverse transcribed into 40 μl of cDNA and diluted with water in ratio 1:1. For each quantitative reverse transcriptase polymerase chain reaction (qRT-PCR) 5 μl of the cDNA preparation was used.

### qRT-PCR for *NPM1* splice variants

For the quantitative measurement of the mRNA expression of *NPM1* splice variants qRT-PCR was performed with the Fast SYBR Green Master Mix (Applied Biosystems, Life Technologies Corporation, Carlsbad, CA, USA) according to the manufacturer's protocol using a 7900HT Fast Real-Time PCR System (Applied Biosystems, Life Technologies Corporation, Carlsbad, CA, USA) in the fast mode. β-actin (*ACTB*) was used as a reference gene. All primers were tested for sensitivity and specificity by conventional PCR and qRT-PCR, resulting in one amplicon of the correct size and one clear peak in the dissociation curve. Samples were assayed in duplicates and reference gene *ACTB* was used for data normalization (see above). Quantity mean values of gene expression were calculated according to the Standard Curve method. For the standard curves, dilution series of cDNA of HeLa cells (for *ACTB*) and Kasumi-1 cells (for *NPM1* splice variants) were used. Data were analyzed using SDS 2.3 software (Applied Biosystems, Life Technologies Corporation, Carlsbad, CA, USA) and visualized using GraphPad Prism 5 (GraphPad Software, La Jolla, CA).

### Western blot analysis

To evaluate the translation of different *NPM1* splice variants, Western blot analysis was performed. Protein extracts from seven cell lines: NB-4 (ACC 207), KG-1 (ACC 14), OCI-AML3 (ACC 582), ME-1 (ACC 537), Kasumi-1 (ACC 220) (DSMZ, Braunschweig, Germany), HEK-293 (ATCC CRL-1573) and K-562 (ATCC CCL-243) (ATCC, Rockville, USA) and CD33+ (CD33 MicroBeads, Miltenyl Biotec) cells from 4 AML patients were run on precast 10% NuPAGE® Bis-Tris Gel (Invitrogen, Life Technologies Corporation, Carlsbad, CA, USA) in MOPS running buffer (Invitrogen, Life Technologies Corporation, Carlsbad, CA, USA), transferred to nitrocellulose membrane (Biorad) and incubated with anti-NPM1 (#3542, Cell Signaling) and anti-β-actin antibodies (#4970,Cell Signaling) or α-tubulin (#2125,Cell Signaling), (β-actin and α-tubulin were used as a loading control). For detection, horseradish peroxidase (HRP)-coupled secondary antibody (#7074, Cell Signaling, Beverly, MA, *USA*) was used followed by chemiluminescence detection using laboratory fresh-made reagent: 100mM TRIS (pH 8,5), luminol (250 mM), coumaric acid (90mM), hydrogen peroxide 30% and visualized with a X-ray film developing machine.

### The quantification of reads in RNA-seq samples

To access the expression of R2 splice variant we analyzed ten transcriptomes of cytogenetically normal AML and FACS-sorted bone marrow samples of three healthy individuals. Bone marrow of healthy donors was sorted by FACS as previously described [35]. Diagnostic AML samples were collected from 10 adult patients with cytogenetically normal karyotype enrolled on German-Austrian AML Study Group (AMLSG) treatment protocols for younger adults [AMLSG 07-04 (NCT00151242)]. Written informed consent was obtained from all patients and healthy donors, and the gene expression study was approved by the IRB.

Total RNA was isolated using AllPrep DNA/RNA Kit (Qiagen). RNA integrity was assessed on Agilent Bioanalyzer using Agilent RNA 6000 Pico or Agilent RNA 6000 Nano Kit (Agilent Technologies) and samples with RNA integrity number (RIN) of at least 7.5 were selected for RNA-seq. A total of 1 μg of RNA was rRNA depleted and sequencing libraries were obtained using Ribo-Zero Gold rRNA Removal Kit (human; Illumina) according the manufacturer protocol. The transcriptomes were sequenced on HiSeq2500 (Illumina) and on average 63.2 million reads were obtained per sample. The reads were aligned to reference genome ucsc.hg19 using STAR aligner [45]. Reads mapping to the unique exon of R2 NPM1 with the coordinates chr5:170833400-170833731 of hg19 were quantified using bedtools with options intersect and split. The counts were RPKM normalized, log2 transformed and plotted in GraphPadPrism software.

### Cell culture and nucleofection

Human embryonic kidney cells (HEK293) (ATCC CRL-1573) and PBMCs from AML patient were cultured at 37°C and 5% CO2 in DMEM/F-12 and RPMI 1640 medium (Biochrom, Berlin, Germany), respectively, supplemented with 10% fetal bovine serum. 1×10^6^ of HEK293 cells were nucleofected using Amaxa Cell Line Nucleofector® Kit V (Lonza Group, Walkersville, MD) and 4 μg of plasmid DNA, strictly according to the manufacturer's protocol. The stable cell lines expressing *NPM1* variants (*NPM1*wt, *NPM1* R2 and *NPM1*mut, described fully in following chapter) fused with fluorescent GFP tag were established in the presence of G-418 (400 μg/ml) in the media for two weeks. The patient cells were collected by centrifugation and resuspended at 8 × 10^6^ cells/100 μl for primary AML cells in the Human B Nucleofector® Kit solution (Amaxa Biosystems, Cologne, Germany). PBMCs were nucleofected with 4 μg of appropriate plasmid using the U-013 program of the Nucleofection Device II (Amaxa Biosystems). The nucleofected cells were cultured at 37°C for 1 day and used for immunofluorescence staining (described in Confocal imaging section).

### Genetic constructs

The GFP-NPM WT expressing plasmid (gift from Xin Wang, Addgene plasmid #17578) [46] were used for creating NPM1 variants (*NPM1*wt, *NPM1* R2 and *NPM1*mut) using site directed mutagenesis. The PCR primers were designed to generate addition or substitution of specific regions in the *NPM1* gene sequence. The sequences of primers used for the mutation were listed in Supplementary Table 1. The PCR products were performed using a high-fidelity polymerase (KOD-Xtreme Hot-start, Millipore; PCR conditions: 1. polymerase activation 94°C 2 min; 2. denaturation 98°C 10 s; 3. annealing 57° 30 s; 4. extension 68° 6 min 45 s). The PCR products were purified by ethanol precipitation followed by DpnI (New England Biolabs, NEB) digestion of the template plasmid.100 ng of the linear PCR product, DpnI-treated and purified on Clean-up columns (A&A) were ligated by Gibson Assembly cloning (NEB) mix following the manufacturers’ protocol. The resulted constructs were introduced into *E.coli* DH10B by electroporation (Eporator, Eppendorf). The single cell colonies growing on kanamycin LB-agar were subjected to colony PCR. Appropriate plasmids were isolated (ExtractMe Plasmid Maxi endotoxin-free kit, Blirt) and verified by sequencing service (Genomed S.A.) using sequencing primers (Supplementary Table 1), and used in transfection/nucleofection procedures.

### Confocal imaging

HEK293 adherent cells were seeded onto eight-well chamber slides (Thermo Scientific™ Nunc™ Lab-Tek™ II Chamber Slide™) and allowed to adhere to the slide (16 hours) before they were fixed with 4% paraformaldehyde or cold (−20°C) methanol. The cells after PBS (Biochrom, Berlin, Germany) washing, were mounted, counterstained with DAPI, and visualized under Nikon Ti confocal microscope (Sendai Nikon Corporation, Miyagi, Japan).

Nucleofected PBMCs grown in suspension were collected, fixed for 20 min in PBS containing 4% parafomaldehyde. The cells were then centrifuged, washed in deionized water and resuspended in 200 μl deionized water. The cells were smeared on adhesion slides (Menzel-Gläser Polysine Slides, Thermo Scientific), dried and washed with water for eliminating the crystals. The cells were then once again washed in PBS, mounted, stained with DAPI and examined by confocal microscopy.

### Statistical analysis

All results are presented as median values. Pairwise comparisons between patient characteristics were performed by Mann-Whitney test for continuous variables and by Fisher's exact test for categorical variables. Survival curves were calculated for overall survival (OS), event-free survival (EFS) and relapse-free survival (RFS) according to Kaplan-Meier and compared using the two-sided long rank test. A Cox model with stratification to account for treatment intensity was used to identify prognostic variables: in addition to *NPM1* splice variants age, WBC count, cytogenetic risk groups, as well as *NPM1* mutations, *CEBPA* double mutations (dm) and *FLT3*-ITD added as explanatory variables in all regression analyses. To provide quantitative information on the relevance of results, 95% confidence intervals (95% CIs) of hazard ratios (HRs) were computed. Statistical analyses were performed using GraphPad Prism 5 (GraphPad Software, La Jolla, CA) and PASW Statistics 18 (SPSS). P-values <.05 were considered to indicate statistical significance.

## SUPPLEMENTARY MATERIALS FIGURES AND TABLES



## Abbreviations

AML: acute myeloid leukemia
CN-AML: cytogenetically normal-AML
NPM1: nucleophosmin-1
FLT3-ITD: FMS-like tyrosine kinase 3-internal tandem duplication
AMLSG: German-Austrian AML Study Group
HVs: healthy volunteers
PBMCs: peripheral blood mononuclear cells
BMMCs: bone marrow mononuclear cells
qRT-PCR: quantitative reverse transcriptase polymerase chain reaction
ACTB: β-actin
OS: overall survival
EFS: event-free survival
RFS: relapse-free survival
95% CIs: 95% confidence intervals
HRs: hazard ratios
MDS: myelodysplastic syndrome).
