# Supplementary material for: Analysis of NPM1 splice variants reveals differential expression patterns of prognostic value in acute myeloid leukemia
Source: Oncotarget. 2017 Aug 3;8(56):95163–75. doi: 10.18632/oncotarget.19871 (PMC5707013; doi:10.18632/oncotarget.19871)
Supplement: Supplementary file 1 [file oncotarget-08-95163-s001.pdf]

Analysis of *NPM1* splice variants reveals differential expression patterns of prognostic value in acute myeloid leukemia

SUPPLEMENTARY MATERIALS

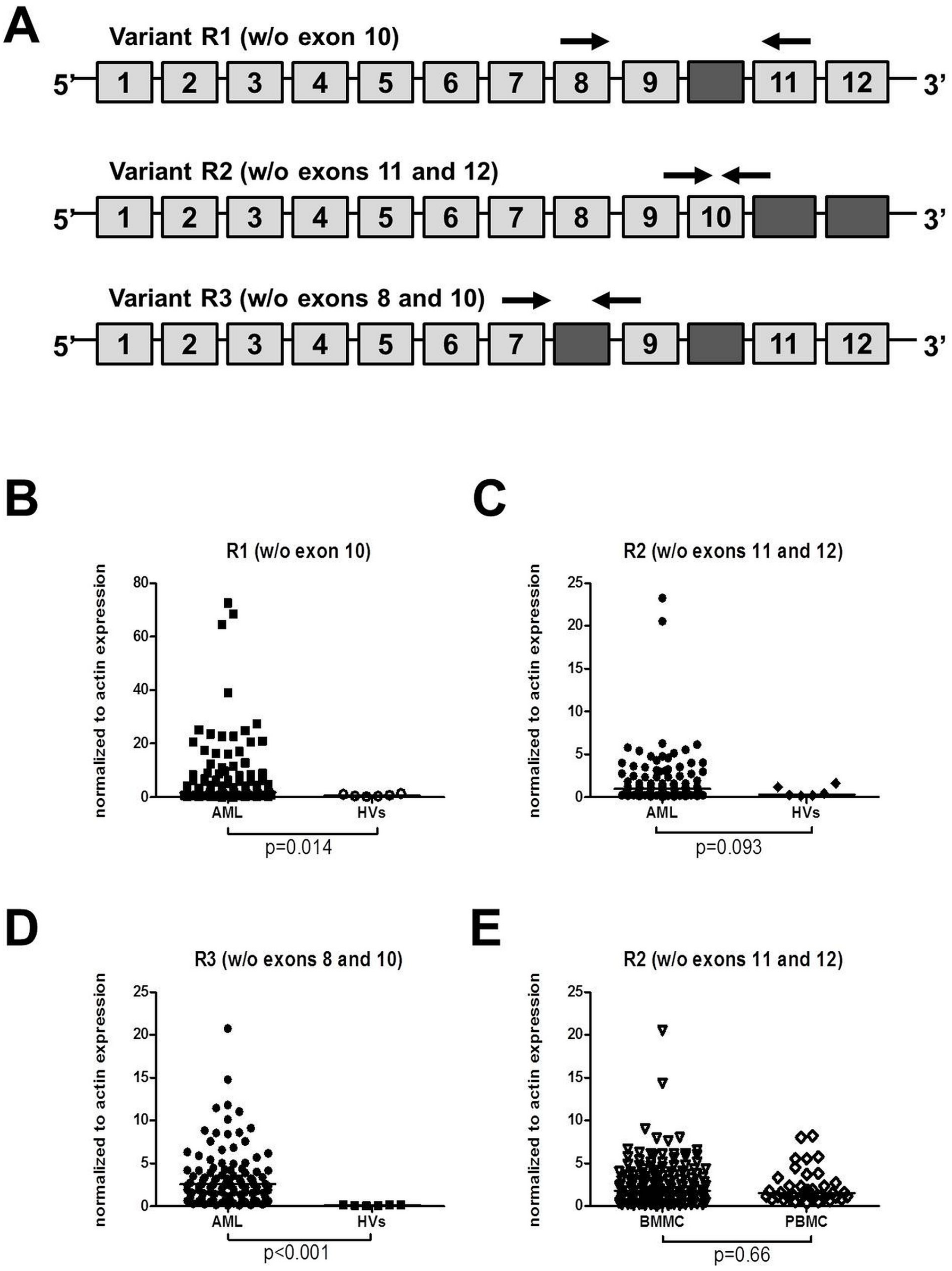

Supplementary Figure 1: (A) A schematic representation of the human *NPM1* gene. For *NPM1* there have been three main splice variants described: R1 translates from exon 1 to 9 and 11 to 12, R2 lacks exons 11 and 12, and R3 does not have exons 8 and 10. **(B-D)** High expression levels of *NPM1* splice variants were observed in AML patients compared to HVs. *NPM1* expression was assessed by quantitative reverse transcriptase PCR (qRT-PCR) and normalized to *ACTB* expression for AML patients and HVs. **(E)** Expression levels of *NPM1* splice variant R2 in bone marrow mononuclear cells (BMMC, n=162) and peripheral blood mononuclear cells (PBMC, n=39) of the entire AML group (n=201).

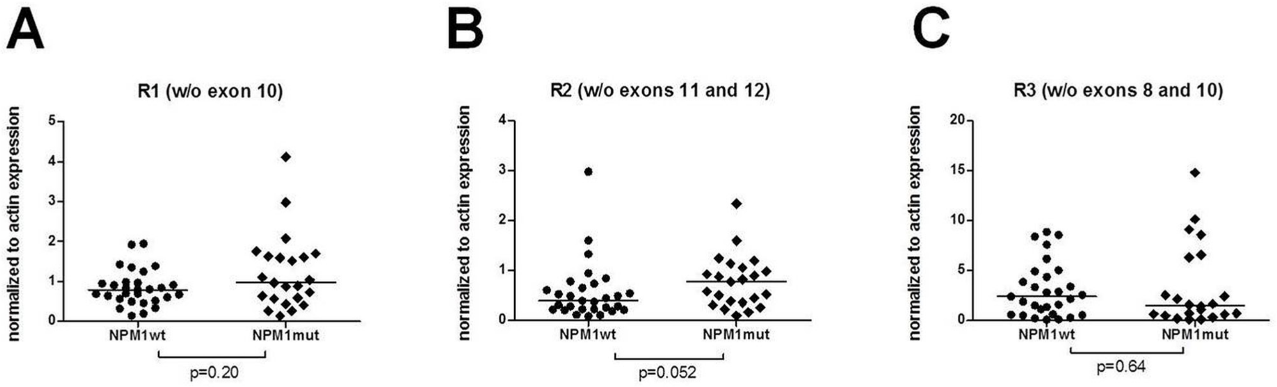

Supplementary Figure 2: Expression levels of *NPM1* splice variants R1, R2 and R3 in first CN-AML cohort (n=52) divided into two groups: with *NPM1* mutations (*NPM1*mut) and without its mutations (*NPM1*wt). **(A, C)** No differences were observed in R1 and R3 expression between *NPM1*mut and *NPM1*wt groups. **(B)** Expression of the R2 splice variant tended to be elevated in *NPM1*mut compared to *NPM1*wt.

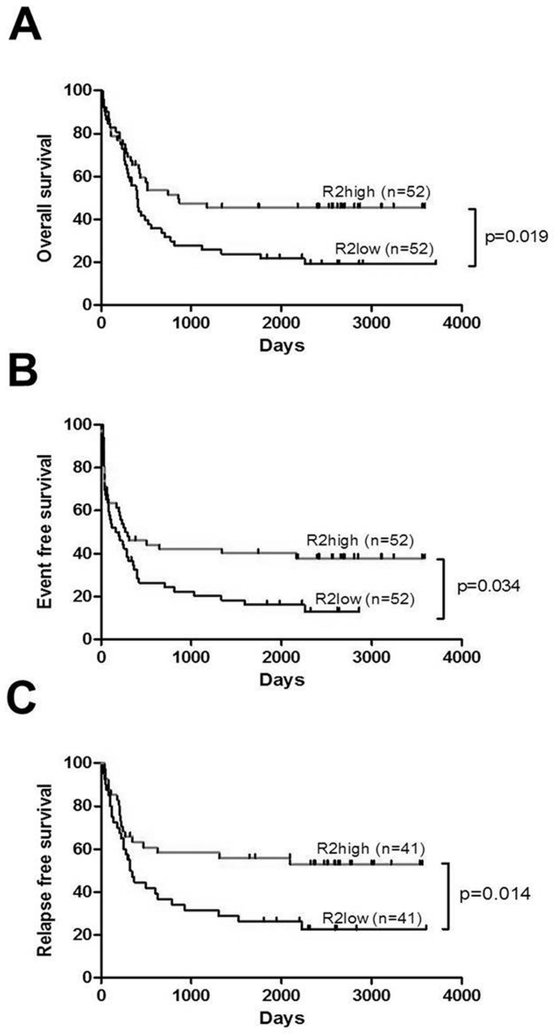

Supplementary Figure 3: (A) OS, **(B)** EFS and **(C)** RFS in the first cohort of 104 AML patients divided according to the expression levels of R2 splice variant.

Supplementary Table 1: Primers used in this study

Construct modification/ purpose	Forward (5´-3´)	Reverse (5´-3´)
Mutation in NPM1 gene (TCTG duplication)	ATCTCTG**<u>TCTG</u>**GCAGTGGA GGAAGTCTCTTTAAGG	TCCACTGC**<u>CAGA</u>**CAGAGAT CTTGAATAGCCTCTTGG
NPM1 R2 splice variant	AAAAAG**<u>CGCAT</u>**TAAGGATC CACCGGATCTAGATAAC	GATCCTTA**<u>ATGCG</u>**CTTTTTC TATACTTGCTTGCATTTTTG
Construct sequencing	AAACTTGCTGCTGATGAAGATG	GCATTCATTTTATGTTTCAGGTTCAGGG

Mutated nucleotides in the original plasmids are shown in bold and the homology regions are underlined.
